# Optimization of gadofosveset intravenous injection scheme for coronary MRA: the pharmacokinetics approach

**DOI:** 10.1186/1532-429X-16-S1-P160

**Published:** 2014-01-16

**Authors:** Mark A Ahlman, Fabio S Raman, Scott R Penzak, Jianing Pang, Debiao Li, David Bluemke

**Affiliations:** 1 Radiology and Imaging Sciences, National Institutes of Health Clinical Center, Bethesda, Maryland, USA; 2 Departments of Radiology and Biomedical Engineering, Northwestern University, Chicago, Illinois, USA; 3 Biomedical Imaging Research Institute, Cedars Sinai Medical Center, Los Angeles, California, USA; 4 Molecular Biomedical Imaging Laboratory, National Institute of Biomedical Imaging and Bioengineering, Bethesda, Maryland, USA; 5 Clinical Pharmacokinetics Research Laboratory, National Institutes of Health Clinical Center, Bethesda, Maryland, USA

## Background

Towards motion-free diagnostic coronary MRA, lengthy (10-15 min) sequences are required. Conventional GBCAs are unable to sustain steady-state intravascular concentrations long enough to maintain low enough T1 signal for good signal/contrast to noise levels[1]. Arterial and venous mixing of contrast takes time that cannot be used for scanning using our free-breathing self-navigated motion-corrected MRA sequence[2,3]. Gadofosveset has a high intravascular residence time and high relaxivity compared to other GBCAs; therefore, we study various injection parameters to accomplish a low T1 time for the longest period of time, as well as a quick equilibrium time with this agent using a dual bolus/slow infusion injection technique.

## Methods

X Normal volunteers without a history of cardiac disease and under the age of 40 were selected for scanning on a Siemens Verio 3T scanner following IRB approved informed consent. Gadofosveset was injected with a dual injection protocol using a Spectris Solaris EP (MEDRAD Inc, Pittsburgh, PA, USA) MR injector using a 70%/%30 and a 60%/40% bolus/slow injection technique. Other variables for injection included the the insertion of a 20-30 second pause between bolus and slow infusion, 1.5 mL/s vs. 3.0 mL/s bolus phase, and single (0.03 mmol/kg) vs double dose (0.06 mmol/kg). Breath-hold, 4-chamber view, single-slice Modified Look-Locker Inversion Recovery (MOLLI) sequences with 11 heart beats were used to measure absolute T1 time in the lumen of the left ventricle using QMass MR ver 7.2 (Medis, Raleigh, NC, USA). Unpaired Mann-Whitney t-testing was used to compare variables for statistical significant difference in medians (p < 0.05).

## Results

**Figure 1:**
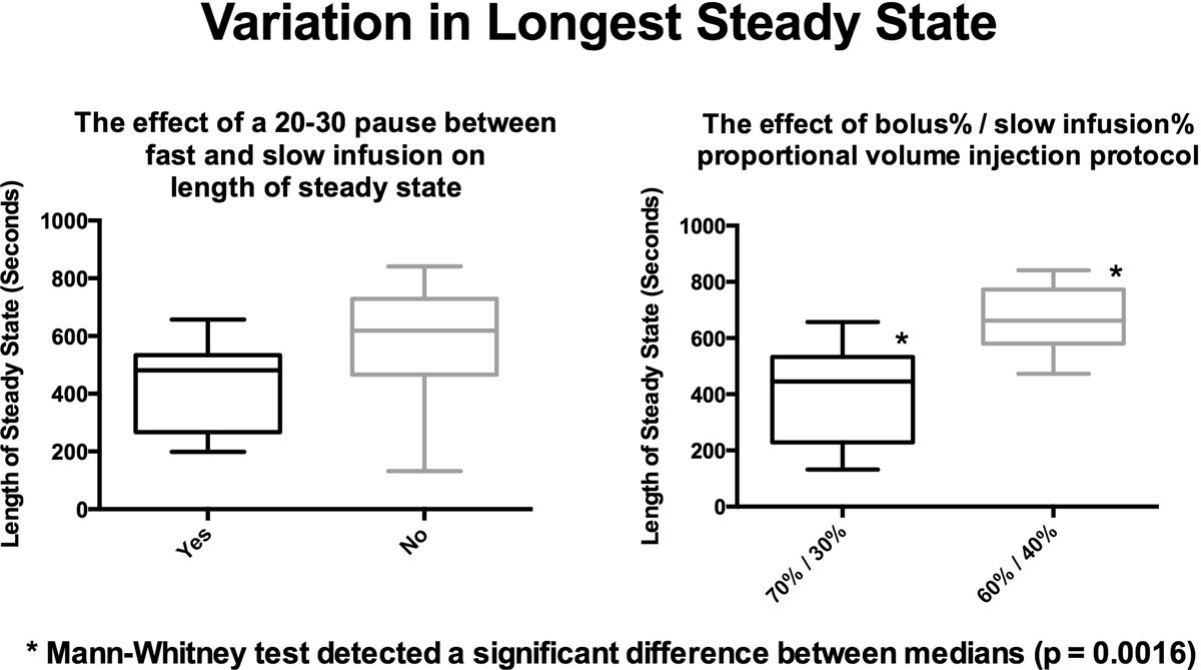


**Figure 2:**
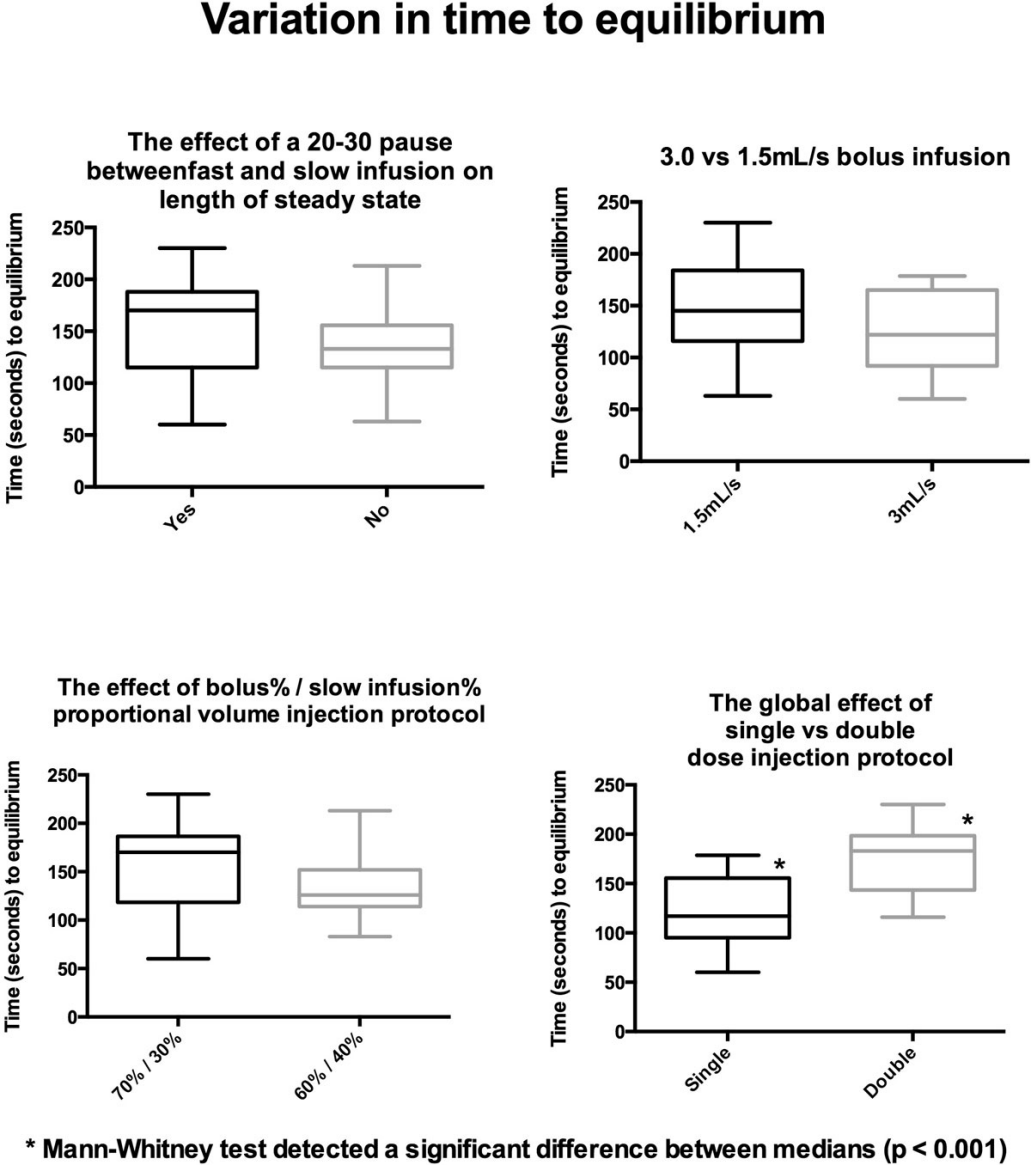


Shown in Figure 1, the only statistically significant parameter that effects the longest intravascular steady state concentration (within 10% variation in T1 time) is the choice of contrast infusion protocol. A 60% bolus followed by a 40% slow infusion results in a median infusion time of >600 seconds. In Figure 2, we see that a double dose regimen has significant bearing on the speed at which intravascular equilibrium is reached. Whereas, the intertion of a pause, the bolus injection rate, and the proportional volumetric (70%/30% vs 60%/40%) bolus/slow infusion protocol do not.

## Conclusions

Using Gadofosveset as a GBCA for self-navigated motion-corrected MRA, we demonstrated how various contrast injection parameters can be used for optimal steady state intravascular concentration for the longest T1 relaxivity. Paradoxically, a double dose protocol increases the time to equilibrium, which does not currently have a clear scientific rationale. Multivariate models are pending, which may elicidate these findings. The length of equilibrium is lengthened by using a protocol that has more contrast in the slow

## Funding

This study is funded by the NIH intramural program.
